# Exploring the Role of ATP-Citrate Lyase in the Immune System

**DOI:** 10.3389/fimmu.2021.632526

**Published:** 2021-02-18

**Authors:** Monica Dominguez, Bernhard Brüne, Dmitry Namgaladze

**Affiliations:** ^1^ Faculty of Medicine, Institute of Biochemistry I, Goethe-University Frankfurt, Frankfurt, Germany; ^2^ Fraunhofer Institute for Translational Medicine and Pharmacology (ITMP), Frankfurt, Germany; ^3^ German Cancer Consortium (DKTK), Partner Site Frankfurt, Frankfurt, Germany; ^4^ Frankfurt Cancer Institute, Goethe-University Frankfurt, Frankfurt, Germany

**Keywords:** acetyl-CoA, ATP-citrate lyase, histone acetylation, macrophage, metabolism

## Abstract

Studies over the past decade have revealed that metabolism profoundly influences immune responses. In particular, metabolism causes epigenetic regulation of gene expression, as a growing number of metabolic intermediates are substrates for histone post-translational modifications altering chromatin structure. One of these substrates is acetyl-coenzyme A (CoA), which donates an acetyl group for histone acetylation. Cytosolic acetyl-CoA is also a critical substrate for *de novo* synthesis of fatty acids and sterols necessary for rapid cellular growth. One of the main enzymes catalyzing cytosolic acetyl-CoA formation is ATP-citrate lyase (ACLY). In addition to its classical function in the provision of acetyl-CoA for *de novo* lipogenesis, ACLY contributes to epigenetic regulation through histone acetylation, which is increasingly appreciated. In this review we explore the current knowledge of ACLY and acetyl-CoA in mediating innate and adaptive immune responses. We focus on the role of ACLY in supporting *de novo* lipogenesis in immune cells as well as on its impact on epigenetic alterations. Moreover, we summarize alternative sources of acetyl-CoA and their contribution to metabolic and epigenetic regulation in cells of the immune system.

## Introduction

The field of immunometabolism, a research area exploring the impact of metabolism on immune responses, gained considerable interest in recent years (1, 2). Numerous studies showed profound changes of metabolism during immune cell activation, such as a rapid increase of glucose utilization in response to antigen recognition in T cells or pathogen sensing in macrophages (3, 4). On the other hand, studies over the last decade uncovered diverse roles of metabolites in regulating immune responses. Thus, metabolites provide substrates for biosynthetic reactions supporting proliferative responses e.g., during T cell expansion (5, 6). Importantly, metabolites can affect transcriptional regulation in the innate and adaptive immune system by epigenetic mechanisms, predominantly through post-translational modifications of histone proteins (7–10). The most studied histone modifications are histone methylation and acetylation. Histone methylation is associated with both transcriptional activation and repression, depending on the histone residue involved. It is regulated by the tricarboxylic acid (TCA) cycle metabolites ketoglutarate, succinate, and fumarate through their impact on the activity of histone demethylases, as well as by the amino acid methionine, providing a precursor for the methyl group donor S-adenosylmethionine (11, 12). Histone acetylation is associated with transcriptional activation, leading to chromatin opening (13). Metabolic control of histone acetylation is predominantly achieved through the acetyl group donor acetyl-CoA (14). In addition, histone deacetylation by a family of sirtuin histone deacetylases is metabolically controlled through their dependence on NAD^+^ (15).

Our review focuses on the role of acetyl-CoA in regulating innate and adaptive immune responses. The key enzyme catalyzing cytosolic acetyl-CoA production is ACLY (16). Here, we summarize the current understanding how ACLY affects the innate and adaptive immune systems, in particular, its roles in modulating metabolic and epigenetic alterations in immune cells. In addition, we discuss alternative sources of acetyl-CoA and their relevance for immune responses.

## Introduction to Acly

ACLY is a ubiquitous homotetrameric enzyme, which is localized in the cytosol and the nucleus (17). It catalyzes the production of cytosolic acetyl-CoA, generating a substrate for *de novo* biosynthesis of fatty acids and cholesterol (16). It also provides acetyl-CoA for acetylation of cytosolic and nuclear proteins, such as histones, thus connecting metabolism with epigenetic control of gene expression (17). In mammals, ACLY expression is particularly high in lipogenic tissues, such as adipose tissue, liver or lactating mammary glands (18). Notably, deletion of the *Acly* gene in mice results in embryonic lethality (19). Moreover, ACLY is known to be highly expressed in many types of cancer cells, supporting proliferation through *de novo* lipogenesis, and making this enzyme a promising target of anti-cancer drugs (20).

**Figure 1:**
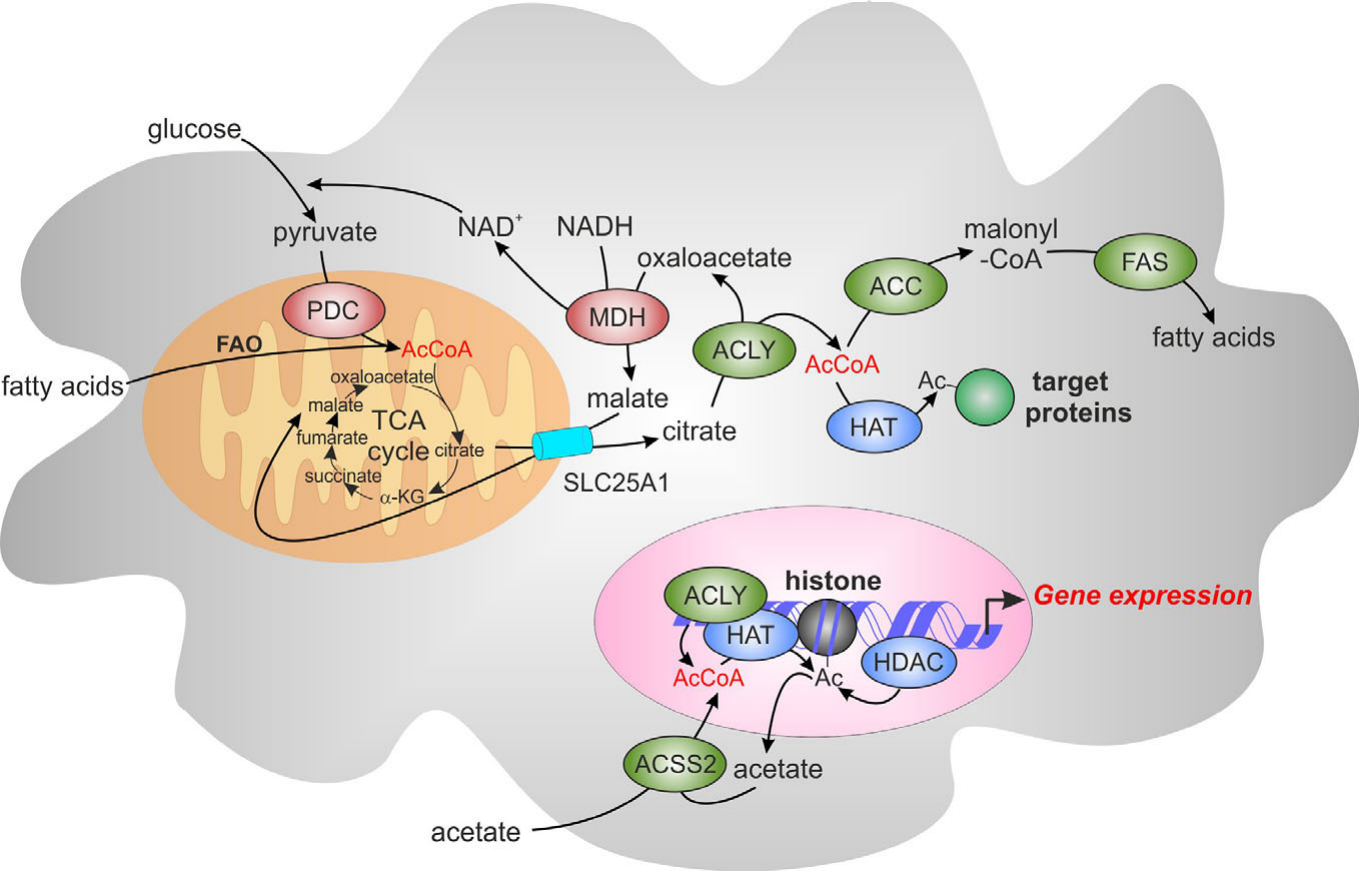
The role of acetyl-CoA and ATP-citrate lyase (ACLY) in *de novo* lipogenesis and epigenetic modifications. Acetyl-CoA (AcCoA) is predominantly produced in mitochondria through oxidation of glycolysis-derived pyruvate by pyruvate dehydrogenase complex (PDC) and through fatty acid oxidation (FAO). AcCoA condensation with oxaloacetate in the tricarboxylic acid cycle (TCA) produces citrate, which can be exported from mitochondria through the mitochondrial citrate carrier, SLC25A1. Citrate is converted by ACLY localized in the cytosol and the nucleus to AcCoA and oxaloacetate. Oxaloacetate is reduced to malate by cytosolic malate dehydrogenase (MDH), providing the source of NAD^+^ to support glycolysis. Malate is imported back into the mitochondria through SLC25A1, completing the citrate-malate shuttle. Cytosolic Acetyl-CoA can be carboxylated to malonyl-CoA by acetyl-CoA carboxylase (ACC). Malonyl-CoA provides a substrate for fatty acid synthase (FAS) for *de novo* fatty acid synthesis. Acetyl-CoA can also be as a substrate of acetylation of cytosolic and nuclear proteins. These reactions are catalyzed by histone acetyltransferases (HATs) whereas histone deacetylases (HDACs) mediate the removal of acetyl group. Acyl-CoA synthetase short-chain family member 2 (ACSS2) offers an alternative source of cytosolic or nuclear acetyl-CoA by employing acetate imported from extracellular sources or recycling acetate from histone deacetylation reactions. α-KG, α-ketoglutarate.

ACLY uses an essential intermediate of the TCA cycle, citrate, as a substrate. Citrate is transported out of the mitochondria predominantly through the mitochondrial citrate carrier, SLC25A1, in exchange for malate (21). In the presence of ATP and CoA, ACLY cleaves citrate to acetyl-CoA and oxaloacetate (22). Acetyl-CoA is then carboxylated to malonyl-CoA by acetyl-CoA carboxylase (ACC), initiating *de novo* fatty acid synthesis. Oxaloacetate is reduced to malate by cytosolic malate dehydrogenase (MDH), providing a source of NAD^+^ to support glycolysis. With SLC25A1-mediated transport of malate back into mitochondria, this cycle is known as the malate-citrate shuttle (23, 24) (**Figure 1**).

ACLY can be phosphorylated at different sites, and phosphorylation can influence its activity. Ser455 has been the first described phosphorylation site, with protein kinase A (25–27) and Akt (28) being major Ser455 kinases. *In vitro* studies have demonstrated that phosphorylation at Ser455 increases the enzymatic activity of ACLY by 6-fold (25). Akt-dependent phosphorylation at Ser455 sustains acetyl-CoA production and histone acetylation upon glucose deprivation (29). Nuclear ACLY is also phosphorylated at Ser455 in response to DNA damage in an ataxia telangiectasia mutated kinase- and Akt-dependent manner, aiding the mechanism of DNA double strand break repair. This process allows spatial and temporal control of acetyl-CoA production, enabling histone acetylation at double strand breaks, recruitment of DNA repair factors and DNA repair by homologous recombination (30). Furthermore, ACLY can be acetylated at lysine residues 540, 546, and 554 (31). Acetylation of ACLY impairs its ubiquitination and degradation. In addition, ACLY is transcriptionally regulated by the sterol regulatory element-binding protein (SREBP)-1 (32, 33).

ACLY is essential for growth and proliferation of cancer cells by supporting membrane biosynthesis through *de novo* fatty acid synthesis (34). Several research groups demonstrated that ACLY knockdown or inhibition arrests the growth of tumor cells both *in vitro* and *in vivo* (35–39). Notably, glucose-dependent cancer cell lines were more sensitive to ACLY knockdown or inhibition because part of the increased proliferation arises from glucose-dependent acetyl-CoA production by ACLY (35). ACLY also participates in nutrient sensing in non-transformed cells. Thus, a knockdown of ACLY impaired glucose-induced insulin secretion in IN832/13 insulinoma cells (40). ACLY was suggested to support the glucose-sensing mechanism by providing the substrate for malonyl-CoA, which then inhibited fatty acid oxidation in favor of glucose oxidation. On the other hand, reduction of ACLY-derived oxaloacetate by MDH provides a source of cytosolic NAD^+^ to support high rates of glycolysis.

Histone acetylation is a dynamic chromatin modification, which is sensitive to acetyl-CoA availability (17). ACLY supports histone acetylation in various cell types, such as adipocytes (17, 41), immortalized fibroblasts (17), skeletal muscle (42), and multiple cancer cell lines (39, 43). Histone acetylation is responsive to changes in glucose availability in an ACLY-dependent manner (17, 29, 44). Nutrient availability influences ACLY expression; ACLY is induced upon high carbohydrate exposure (45) and repressed during high fat feeding (46). In high-fat diet-fed mice, acetyl-CoA abundance and histone acetylation markers were decreased in white adipose tissue and pancreas but not in the liver, likely as a consequence of ACLY suppression (47). This interpretation is reinforced by the findings that acute *Acly* deletion from mature adipocytes *in vitro* suppresses both acetyl-CoA levels and histone acetylation (47). On the other hand, mesangial cells exposed to high glucose conditions increased histone H3 acetylation at Lys9/Lys14 and Lys18, inducing regulation of potent fibrotic factors, such as transforming growth factor-β1, -β3, or connective tissue growth factor. This was achieved by upregulation and nuclear translocation of ACLY, providing the substrate for histone acetylation and thus, influencing epigenetic regulation of diabetic renal fibrosis (48). Taken together, these findings indicate that diet has the ability to impact histone acetylation and gene expression programs, potentially giving a predisposition to metabolic diseases and cancer, which could be targeted through ACLY.

Due to its impact on cancer cell growth, ACLY is an attractive target for anti-cancer drug development efforts. Several effective ACLY inhibitors have been described, such as BMS 303141 (49), SB 204990 (50), Medica 16 (51), hydroxycitrate (52), and, recently, NDI-091143 (53); some of them being able to reduce tumor growth *in vivo* (35). Another therapeutic area where ACLY inhibitors have been actively investigated is cardiovascular disease. In particular, bempedoic acid (ETC-1002), is a liver-targeting ACLY inhibitor with promising lipid-lowering properties. The very long chain acyl-CoA synthetase-1 (gene name *SLC27A2*), which is predominantly expressed in the liver and kidney, converts bempedoic acid to its active –CoA derivative (18). As a consequence, cholesterol *de novo* biosynthesis in the liver is inhibited. Bempedoic acid showed clinical efficacy to reduce low-density lipoprotein cholesterol in hypercholesterolemic patients taking statins or in statin-intolerant patients in recent trials (54, 55). It is now approved by the U.S. Food and Drug Administration for the treatment of adults with heterozygous familial hypercholesterolemia or established atherosclerotic cardiovascular disease that require additional lowering of low-density lipoprotein cholesterol.

In addition to ACLY, another important enzyme having the ability to produce acetyl-CoA outside of the mitochondria is acyl-CoA synthetase short-chain family member 2 (ACSS2) (56). This enzyme produces acetyl-CoA using acetate imported from extracellular sources or recycling acetate from histone deacetylation reactions (44, 57–60). Several cancer cell lines, particularly under conditions of ACLY deficiency, hypoxia or nutrient limitation, favor this pathway (57, 59), but even under glucose-replete conditions ACSS2 may be preferably used to support *de novo* lipogenesis, as in case of human cytomegalovirus infection (HMCV) (61). ACSS2 may directly bind to promoter regions of lysosomal and autophagy genes in glioblastoma cells or to regulatory regions of neuronal genes in hippocampal neurons to locally produce acetyl-CoA supporting histone acetylation and gene expression (62, 63).

Likewise, the pyruvate dehydrogenase complex (PDC) is also described to be present in nucleus and generate acetyl-CoA for histone acetylation, e.g., during cell cycle progression (64, 65).

## Acly in the Innate Immune System

The innate immune system is mainly comprised of hematopoietically derived cells, including monocytes, macrophages, natural killer (NK) cells, innate lymphoid cells, neutrophils, mast cells, basophils, eosinophils, and dendritic cells (DC). Innate immune cells express a range of germline-encoded pattern recognition receptors (PRR). Upon infection or injury, PRR recognize highly conserved pathogen-associated or damage-associated molecular patterns, such as bacterial lipopolysaccharides (LPS) or heat shock proteins, causing rapid and profound alterations in metabolism and gene expression (66). Recent studies highlighted ACLY as an important player controlling lipid metabolism and histone acetylation during innate immune responses.

### The Role of ACLY in Macrophage Polarization

Macrophages respond to environmental cues such as bacterial or viral infections, nutrients, or cytokines by remodeling their transcriptome, and consequently, phenotype (67). This response, referred to as macrophage polarization, was historically characterized as pro-inflammatory M1 polarization triggered by LPS and interferon γ (IFNγ) *vs*. M2 polarization triggered by interleukin (IL)-4 or IL-13, leading to an anti-inflammatory profile, enabling tissue repair and remodeling (68). Whereas M1 macrophages switch their metabolism from oxidative phosphorylation (OXPHOS) to glycolysis (66), M2 macrophages meet their energetic requirements mainly through OXPHOS (69).

First studies identifying ACLY as an essential mediator of macrophage inflammatory responses initially focused on the export of citrate from mitochondria to the cytosol *via* tricarboxylic acid carrier SLC25A1 (21, 70), later showing the key involvement of ACLY (71). Thus, human primary macrophages upregulated ACLY protein and mRNA upon activation with the pro-inflammatory stimuli LPS, IFNγ, and tumor necrosis factor α (TNFα). In the U937 monocytic cell line, pharmacological inhibition or mRNA silencing of ACLY caused a reduction in nitric oxide (NO), reactive oxygen species (ROS), and prostaglandin E_2_ (PGE_2_) release upon LPS stimulation. While acetyl-CoA production and *de novo* synthesis of arachidonic acid were postulated to underlie ACLY-dependent prostaglandin production, generation of oxaloacetate and its conversion to malate and finally to pyruvate by malic enzyme were suggested to provide NADPH to support NO and ROS production (71). Whereas no studies directly addressed the impact of ACLY on *de novo* lipogenesis in macrophages, ACLY knockout THP-1 monocytic cells exhibited delayed cell growth (72), likely due to deficiencies in *de novo* lipogenesis, as previously observed in other cancer cell lines (34, 73, 74). In the context of macrophage anti-bacterial activity, however, targeting *de novo* lipogenesis by using a myeloid-specific knockout of ACC was dispensable for macrophage and DC responses to *Mycobacterium tuberculosis* (75).

ACLY-dependent lipid biosynthesis appears to support immature myeloid cell proliferation. Inhibiting ACLY or fatty acid synthase (FAS) in BN murine fetal liver cells abolished their proliferation, which could be rescued by acetyl-CoA supplementation. Incorporation of exogenously added radiolabeled acetyl-CoA into cellular lipids increased in ACLY-inhibited cells, confirming the contribution of ACLY to *de novo* lipogenesis in myeloid progenitors (76). *De novo* lipogenesis was also increased in bone marrow-derived mouse macrophages (BMDM) treated with a toll-like receptor (TLR) 9 agonist CpG oligodeoxynucleotide, which was postulated to promote membrane fluidity and increase the capacity of macrophages to phagocytize tumor cells. Inhibiting ACLY with BMS 303141 abolished increased tumor cell phagocytosis in CpG-treated macrophages (77).

The impact of ACLY on macrophage M1 (78, 79) *vs*. M2 (80) polarization has recently been reported using BMDM. The production of acetyl-CoA supports both pro- and anti-inflammatory functions of macrophages through various mechanisms (78, 80). LPS-stimulated macrophages increased glycolysis and oxygen consumption within 2 h followed by a decrease in oxygen consumption at 24 h (78). The early response to LPS was characterized by elevated metabolites of the Krebs cycle such as succinate, lactate, and citrate, the latter fueling ACLY-dependent synthesis of acetyl-CoA. In addition, the authors observed Akt-dependent phosphorylation of ACLY at Ser455, which was abolished in macrophages deficient for the TLR signaling adaptors MyD88 and TRIF. Thus, an increased glycolytic flux to citrate and ACLY activation, were suggested to support increased cytosolic acetyl-CoA generation in LPS-stimulated macrophages. Pharmacological ACLY inhibition using BMS 303141 reduced mRNA expression of secondary response genes *il6* or *il12b* without having any effect on primary response genes, such as *cxcl1* and *il1b* in LPS-treated BMDM. This correlated with the reduction of histone H3 acetylation at Lys27 and total H4-acetylation, and decreased chromatin accessibility at *il12b* locus upon ACLY inhibition. Mass spectrometry analyses using C13-labeled glucose confirmed ACLY-dependent incorporation of the label into Lys9, Lys14, Lys18, Lys23, and Lys27 acetylated lysine residues of histone H3, which was increased upon LPS treatment (78). Some residual histone acetylation may be due to acetate recycling through ACSS2 as previously described in other models (57). Overall, this shows that increased histone acetylation in LPS-stimulated BMDM is a direct result of glucose metabolism and ACLY-dependent acetyl-CoA production. Interestingly, global analysis of gene expression revealed increased mRNA expression of anti-inflammatory cytokines IL-10 as well as IL-1 receptor antagonist in LPS-stimulated macrophages upon ACLY inhibition. Whether this is associated with changes in histone acetylation at regulatory elements of corresponding genes remains to be established (78). Translating the results obtained using BMDM to *in vivo* model of LPS-induced peritonitis, Lauterbach and colleagues confirmed the importance of ACLY for pro-inflammatory macrophage polarization. Thus, mice co-injected with BMS 303141 revealed decreased protein levels of IL-6 and IL-12p70 in the peritoneum and serum, suggesting that ACLY inhibition was able to alter the local and systemic inflammatory profiles (78).

Similarly, Langston et al. observed increased ACLY Ser455 phosphorylation in LPS-treated BMDM. Inhibiting ACLY using SB 203580, BMS 303141, or Medica 16 reduced LPS-triggered histone H3 Lys27 acetylation at *il6* and *il1b* promoters, and attenuated the expression of these genes. Early in the activation course (up to 3 h), LPS increased glucose oxidation and glucose flux into acetyl-CoA, which was supported by the activity of the glycerol-phosphate shuttle system. Disrupting this shuttle by knocking out mitochondrial glycerol 3-phosphate dehydrogenase (Gpd2) prevented glucose conversion to acetyl-CoA, histone acetylation at *il6* and *il1b* promoters, and *il6* and *il1b* mRNA expression in LPS-stimulated macrophages (79). In contrast, prolonged LPS stimulation (12-24 h) reduced glucose oxidation and acetyl-CoA generation upon restimulation, and prevented inflammatory gene induction, a condition known as LPS tolerance. Interestingly, induction of LPS tolerance depended on glucose metabolism and Gpd2 activity, probably by inducing reverse electron flow to complex I of the mitochondrial respiratory chain. This resulted in suppressed NADH oxidation and TCA cycle flux.

ACLY was also implicated in the regulation of alternative macrophage activation by Th2 cytokine IL-4. Analyzing IL-4-stimulated BMDM, Covarrubias et al. observed increased fatty acid oxidation and OXPHOS in accordance with previous studies in this system (81). At the same time, IL-4-treated macrophages displayed an increase in glycolysis, which was dependent on the Akt kinase. Furthermore, metabolic flux analyses revealed contributions of glucose, glutamine, and fatty acids to acetyl-CoA production upon IL-4-stimulation. The authors showed IL-4 to promote ACLY phosphorylation at Ser455 in an Akt-dependent manner. ACLY inhibition attenuated histone H3 and H4 acetylation at promoters of Akt-dependent IL-4 target genes, which correlated with attenuated mRNA expression of these genes in macrophages treated with ACLY inhibitor. Thus, ACLY supported macrophage response to IL-4 through acetyl-CoA provision for histone acetylation in a gene-specific manner (80). What drives this specificity remains to be investigated.

Recent findings in myeloid-specific ACLY knockout mice challenge its role supporting pro-inflammatory macrophage polarization (82). Contrarily, LPS-induced IL-6, TNF, NO, and ROS were increased in ACLY-deleted macrophages. Conversely, IL-4 treated ACLY-knockout macrophages showed similar results to Covarrubias et al., with reduced gene expression of Akt-dependent genes such as *Arg1* and *Retnia.* It was hypothesized that the reduction in response to IL-4 in ACLY-deleted macrophages is due to lower H3 Lys27 acetylation. However, no differences in histone acetylation were noticed between wild-type and ACLY-deficient LPS-treated macrophages (82).

**Figure 2:**
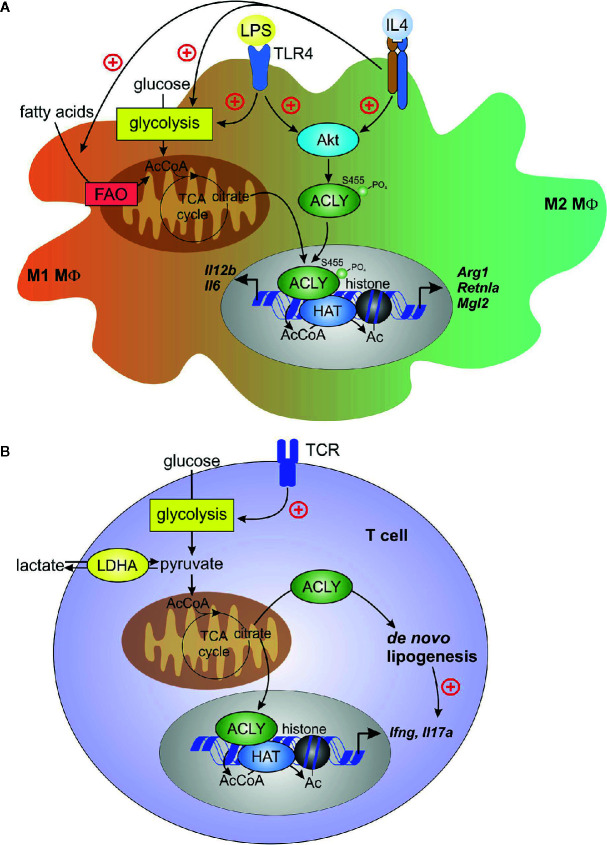
ACLY in macrophages and T cells. **(A)** Macrophage pro-inflammatory (M1) activation by toll-like receptor 4 (TLR4) ligand lipopolysaccharide (LPS) or alternative (M2) activation by interleukin (IL)-4 induces Akt-dependent phosphorylation and activation of ACLY. TLR4 activation enhances glycolysis and, in the early phase of inflammatory response, increases TCA cycle flux to citrate. citrate translocates to cytosol and the nucleus for ACLY-dependent acetyl-CoA (AcCoA) formation. In IL-4-stimulated cells, both fatty acid oxidation (FAO) and glycolysis contribute to AcCoA generation. ACLY-dependent AcCoA provides substrate for histone acetylation, which supports increased expression of pro-inflammatory genes (*il6, il12b*) or IL-4 target genes (*Arg1, Retnla, Mgl2*). **(B)** T-cell receptor ligation during activation of effector T cells increases glycolysis, thereby enhancing pyruvate to lactate conversion by lactate dehydrogenase (LDH). This conversion promotes citrate exit from the TCA cycle, driving cytosolic AcCoA generation by ACLY. Under inflammatory conditions, extracellular lactate may enter T cells and feed to TCA cycle, providing a source of citrate. ACLY-derived AcCoA supports *de novo* lipogenesis in activated T cells, and provides substrate for histone acetylation, which is necessary for increased expression of *ifng*. *De novo* lipogenesis promotes *il17a* expression in activated T_H_17 cells.

The role of ACLY in polarization of human macrophages remains less understood. BMS 303141 inhibited LPS-stimulated *IL6* and *IL12B* mRNA expression in primary human macrophages, corroborating data obtained in BMDM (78). In human primary macrophages stimulated with IL-4, ACLY inhibitors reduced the expression of IL-4 target genes, but H3 histone acetylation at Lys27 and Lys14 was not influenced. ACLY knockdown affected neither IL-4 target gene expression nor histone acetylation. Furthermore, in ACLY knockout THP-1 cells, differentiated into macrophages, ACLY inhibitors still reduced IL-4-induced target gene expression, suggesting off-target effects of these inhibitors. Collectively, these data suggested that IL-4 polarization in human primary macrophages did not require ACLY (72). Nevertheless, further studies are required to define roles of ACLY in M1 *vs*. M2 polarized human macrophages. **Figure 2A** summarizes current concepts on the role of ACLY in macrophage polarization.

So far only a few studies investigated the role of acetyl-CoA in macrophages in pathology. In pathologic retinal angiogenesis, perivascular macrophages upregulate glycolysis, with concomitant increases of acetyl-CoA production and histone acetylation at promoters of pro-angiogenic genes, which was sensitive to the deletion of glycolytic activator 6-phosphofructo-2-kinase/fructose-2,6-bisphosphatase (77). Acetyl-CoA flux into the mevalonate pathway supported pro-fibrotic polarization of macrophages in response to lung injury (83).

A recent study focused on the role of ACLY in pathology of atherosclerotic disease (82). Following observations showing a marked increase in phosphorylated ACLY in macrophage-rich, rupture-prone areas of human atherosclerotic plaques, the authors investigated the impact of macrophage ACLY deletion *in vivo* on atherosclerotic disease progression in low-density lipoprotein receptor knockout mice. Myeloid ACLY deficiency enabled stable plaque formation characterized by increased collagen deposition and fibrous cap, along with a smaller necrotic core. Whereas the advantageous plaque phenotype observed in ACLY knockout macrophage transplanted mice could not be explained by changes in macrophage polarization, deregulation of fatty acid and cholesterol biosynthesis were noticed. Thus, ACLY-knockout macrophages showed increased expression of genes involved in fatty acid and cholesterol uptake, and a reduction of liver X receptor (LXR) target genes involved in cholesterol efflux, apparently reflecting the response to a decreased supply of acetyl-CoA for *de novo* fatty acid and cholesterol biosynthesis. Moreover, ACLY*-*deficient macrophages were more prone to undergo apoptosis and more efficient at efferocytosis, giving an explanation for the smaller necrotic core and plaque stabilization. Taken together, these findings indicate that targeting ACLY in macrophages could be advantageous to reduce atherosclerotic disease progression.

The discrepancies that arise when comparing the studies on the role of ACLY in macrophage polarization cannot be disregarded. A possible explanation for these differences could be the use of distinct experimental settings. In addition, these findings also show that pharmacological inhibition, knockdown, or constitutive knockout of ACLY can give very distinct results (72, 78, 82), highlighting the need for critical evaluation when interpreting the data. Furthermore, M1/M2 polarization data *in vitro* might not reflect the real complexity of the *in vivo* setting, where macrophages are simultaneously exposed to different environmental signals. Apparently, further *in vivo* studies may reveal more nuanced roles of ACLY and ACLY-dependent lipogenesis and epigenetic modulation in supporting macrophage phenotypic plasticity.

A number of studies have recognized ACCS2 as being relevant in modulating inflammatory responses in innate immune cells. For instance, comparison of monocyte transcriptomes of two different rat strains in response to obesogenic diet identified ACSS2 as a hub gene in a nutrient-sensing transcriptional network (84), with inverse correlation of ACSS2 expression levels and plasma glucose, triglyceride, leptin and non-esterified fatty acids as well as body weight and total fat weight. Monocyte ACSS2 may also have relevance in the context of acute alcoholic hepatitis (85). Ethanol-exposed monocytic MonoMac6 cells convert it to acetate, which in turn is metabolized to acetyl-CoA, resulting in increased histone acetylation and the production of pro-inflammatory cytokines IL-6, IL-8, and TNFα, which was sensitive to the double knockdown of ACSS1 and ACSS2. On the other hand, acetate and ACSS2 appears to have a neuroprotective function in the brain. Thus, acetate supplementation (in the form of glyceryl triacetate) has been shown to attenuate neuroinflammation *in vivo* (86, 87) and in microglia *in vitro* (88) by altering histone acetylation and reducing pro-inflammatory cytokine production.

Interestingly, histone lactylation has recently been described as a novel type of metabolic regulation of gene expression in LPS-stimulated macrophages, which does not depend on ACLY. Histone lactylation in M1-polarized macrophages was suggested to serve as a mechanism to initiate homeostasis in the late phase of inflammatory response. Therefore, its role differs from histone acetylation that enables the induction of pro-inflammatory genes (89).

### Metabolic Regulation and Epigenetic Control of DC by Acetyl-CoA

DC are highly specialized antigen presenting cells with a fundamental role at initiating and maintaining adaptive responses (90). TLR agonists instantly increase glycolysis to support the anabolic demands of DC activation, causing a metabolic switch from OXPHOS. It seems that rapid increase of glycolysis aims to provide citrate supporting *de novo* lipogenesis for DC activation (91). Although pharmacological inhibition of FAS had no effect on mRNA expression of pro-inflammatory cytokines, it, as well as silencing of SLC25A1, inhibited protein expression of IL-6, IL-12, and TNFα as well as PGE_2_ production in LPS-stimulated DC. *De novo* lipogenesis seems to contribute to rapid expansion of the endoplasmic reticulum and Golgi apparatus in LPS-stimulated DC, to increase cytokine synthesis and secretion. Another study focusing on the effects of the mitochondrial chaperone p32 (or complement C1q binding protein) in DC, found that *de novo* lipogenesis following LPS stimulation is essential for DC maturation (92). p32-deficient DC showed reduced PDC activity, which decreased citrate levels upon LPS stimulation. These cells also exhibited reduced protein expression of mitochondrial complex I and IV, associated with impaired mitochondrial respiration and a compensatory switch to glycolysis. Although p32-deficient DC had intact cytokine responses to LPS or a TLR9 agonist, they displayed reduced surface expression of maturation markers, such as CD40, CD86, or MHC class I and II. DC maturation was sensitive to reduction of PDC expression, but not to inhibition of respiratory chain complexes I or V. p32-deficient cells had attenuated ACLY Ser455 phosphorylation in response to LPS or TLR9 activation. LPS-induced lipid accumulation was also reduced in p32^−/−^ DC. C75, an inhibitor of FAS, attenuated the acquisition of DC maturation markers. Together, these studies indicated the role of ACLY-dependent *de novo* lipogenesis in DC activation and maturation, but direct evidence for an ACLY involvement is still lacking (92).

Although not directly focused on the effects of ACLY, Marquez et al. claimed that human monocyte-derived DC rely on mitochondrial pyruvate entry to support the induction of *IL10* and *IL23A* mRNAs by zymosan or LPS. Induction of *IL23A* driven by LPS, but not zymosan, and *IL1B* induction by both stimuli were reduced upon ACLY inhibition by BMS 303141, whereas *IL10* and *TNF* induction remained unaffected. However, ACLY inhibition left acetylation at the nuclear factor-κB-responsive site of *IL23A* promoter unaffected, although this acetylation was sensitive to inhibition of pyruvate entry (93). This could support the idea that ACLY is not the sole player in acetylation (17); however, acetate supplementation did not restore cytokine mRNA expression in cells not allowing pyruvate entry. Based on experiments in bone marrow-derived DC from mice deficient in platelet activating factor (PAF) receptor, the authors suggested that acetyl-CoA supported autocrine PAF generation by DC, but direct evidence for this was lacking.

### The Role of ACLY in NK Cells

NK cells are innate lymphocytes, which exert powerful anti-tumor and anti-viral responses. Although traditionally classified as innate cells, they also have important functions in aiding the adaptive response. Increased energy requirements to properly kill tumor and virus-infected cells require a metabolic switch from OXPHOS to glycolysis (94).

A recent study identified SREBP transcription factors to modulate glucose metabolism of activated NK cells in part through increased gene expression of SLC25A1 and ACLY. Activated NK cells increased levels of glucose-derived citrate, which, instead of being processed in the TCA cycle, contributed to ACLY-dependent cytosolic acetyl-CoA generation. ACLY-derived oxaloacetate was suggested to support cytosolic NAD^+^ generation by the citrate-malate shuttle. As evidence for importance of this mechanism, inhibiting ACLY by BMS 303141 attenuated both glycolytic and oxidative metabolism of activated NK cells. Moreover, cytokine-stimulated NK cells treated with ACLY inhibitor displayed reduced IFNγ production and lower granzyme B expression, suggesting a role for ACLY in NK cell function. Whereas the ability of ACLY to provide acetyl-CoA for histone acetylation was not directly addressed in that study, it was suggested to underlie the observed effects of ACLY inhibitors on IFNγ production. At the same time, inhibiting fatty acid synthesis was without effect on proliferation or effector function of activated NK cells, suggesting that ACLY-dependent *de novo* lipogenesis is of little importance in this system (23).

## Acly in the Adaptive Immune System

In contrast to the innate immune system, adaptive immunity develops over a host’s lifetime and involves the production of antibodies against a pathogen. T and B lymphocytes are the key players of adaptive immunity. These cells express unique receptors and following an initial encounter with a foreign antigen, undergo clonal expansion. As a result, the adaptive immunity is highly specific but requires time to develop. Some of the adaptive immune cells develop into memory cells and regulatory cells, which control the initiation and development of the immune response and inflammation (95). Although not extensively reported to date, ACLY has also been shown to impact certain adaptive immune responses through metabolic and epigenetic remodeling.

### The Role of ACLY in T Lymphocytes

To develop from naïve T cells into the different T cell lineages, T cells must undergo a considerable metabolic switch in order to meet the demands of cell growth and division (1). While mitochondrial metabolism is utilized by naïve T cells, activated T cells change their metabolism from OXPHOS to glycolysis, which is required to sustain a rapid effector response to stimuli (96, 97). This metabolic shift also supports the inflammatory profile of activated T cells through enhanced expression of cytokines such as IFNγ and IL-17A (98). T cells have a great capacity to synthesize essential biomolecules supporting biomass increase during rapid proliferation. *De novo* fatty acid synthesis linked to glucose metabolism is essential to support activation-induced differentiation of CD8^+^ effector T cells or T_H_17 cells. Thus, T cells cultured under T_H_17-inducing conditions upregulated ACLY, suggesting enhanced activity of *de novo* lipogenesis pathway (99). Whereas the role of ACLY was not addressed in this study, pharmacological inhibition or genetic deficiency of ACC reduced the IL-17 expression and proliferation of T_H_17 cells (100). Similarly, T_H_17 differentiation was attenuated by inhibiting FAS. Interestingly, *de novo* lipogenesis in T_H_17 cells could also be fueled by high levels of extracellular lactate, often occurring in highly inflamed tissues (7). Inhibiting ACC reduced IL-17 production in lactate-exposed cells. Furthermore, mice with a T cell-specific knockout of ACC display reduced numbers of peripheral CD8^+^ T cells (101), which was attributed to decreased survival of these cells, while maintaining intact T cell effector functions. Together, these studies indicate the importance of *de novo* lipogenesis for effector T cell expansion.

The ability of ACLY-produced acetyl-CoA to support effector T cell responses *via* histone acetylation became evident from several recent studies. Notably, acetyl-CoA was important for IFNγ production by activated T_H_1 cells (9). Lactate dehydrogenase A (LDHA) is induced in activated CD4^+^ T cells to maintain aerobic glycolysis. LDHA-deficient T cells displayed reduced cytosolic acetyl-CoA and impaired histone H3 Lys9 acetylation at the IFNγ promoter and enhancer loci. It was argued that in the absence of LDHA, more acetyl-CoA was consumed in the TCA cycle, leaving less citrate available for cytosolic export to provide a substrate for ACLY. The role of ACLY in this system was confirmed by observations that its inhibition reduced IFNγ production (9). A direct genetic proof of the mitochondrial citrate export involvement in driving T cell epigenetic changes was obtained recently. In CD4^+^ T cells cultured under T_H_1 conditions, CRISPR-Cas9-mediated deletion of *Acly* and *Slc25a1* resulted in attenuated IFNγ production, reduction of total histone H3 Lys9 acetylation, and reduced proliferation (102). Together, these data indicate that ACLY, through the production of cytosolic acetyl-CoA derived from mitochondrial citrate export, largely influences proliferation and transcriptional remodeling during T_H_1 cell activation (summarized in **Figure 2B**).

Acetyl-CoA generation by ACLY also contributes to epigenetic regulation of CD8^+^ T cells. Thus, ACLY was shown to mediate the conversion of citrate derived from mitochondrial pyruvate oxidation to acetyl-CoA in CD8^+^ effector memory T cells. This in turn was essential for rapid IFNγ production during a recall response (103). It was speculated that ACLY-generated acetyl-CoA could be used as a substrate for histone acetylation at genetic loci relevant for T cell effector function, including the IFNγ locus (103). Interestingly, ACLY was also necessary for IFNγ production in CD8^+^ memory T cells in response to exogenous acetate (104). Whereas in cancer cell lines acetate is converted to acetyl-CoA by the action of cytosolic ACSS2, this enzyme was dispensable for acetyl-CoA generation in acetate-treated memory T cells. Instead, the authors proposed the conversion of acetate into acetyl-CoA by mitochondrial ACSS1, with subsequent processing in the TCA cycle, export of citrate into cytosol, and its breakage by ACLY. This process occurred despite glucose availability, and increased ACLY-derived cytosolic acetyl-CoA promoted acetylation and activation of glyceraldehyde 3-phosphate dehydrogenase, thus, enhancing glycolysis and boosting rapid memory responses (104). Acetate could also support histone acetylation and IFNγ production in CD8^+^ T cells under conditions of glucose restriction, mimicking the tumor microenvironment (105). Global analyses of histone H3 Lys9 acetylation and chromatin accessibility showed that acetate supplementation caused widespread increases of acetylated histone H3 Lys9 and chromatin opening in glucose-restricted T cells. Among the upregulated genes in acetate-supplemented cells, some, such as *ifng*, granzymes, *tnf*, and *Tbx21*, were associated with enhanced effector responses. Acetate supplementation *ex vivo* also increased IFNγ production by tumor-infiltrating lymphocytes. In glucose-restricted cells, ACSS2 silencing was able to inhibit acetate-driven increases of IFNγ expression and histone acetylation. Finally, ethanol-derived acetate may underlie anti-inflammatory effects of ethanol consumption in autoimmune arthritis by suppressing activation of follicular T helper cells (106).

Intracellular acetyl-CoA levels have also been recently shown to regulate T cell fate in the context of the tumor microenvironment (107). Necrotic tumor areas are characterized by high levels of interstitial K^+^. This was shown to inhibit the uptake of nutrients in T cells present in these areas, creating a state of functional caloric restriction, characterized by elevated autophagy and increased reliance on mitochondrial respiration. Although mitochondrial acetyl-CoA increased in high K^+^-exposed CD8^+^ T cells, nucleocytosolic levels of citrate and acetyl-CoA dropped, causing reduced histone H3 Lys9 acetylation at loci associated with T cell activation and exhaustion (*Ifng, Pdcd1, Cd244*) with impaired expression of corresponding genes. At the same time, high K^+^ conditions preserved the expression of stemness genes. These effects were mimicked by inhibiting ACLY using hydroxycitrate and reversed upon provision of acetate or electroporation of acetyl-CoA. Treating tumor antigen-specific CD8^+^ T cells with hydroxycitrate prior to adoptive transfer to tumor-bearing mice increased *in vivo* T cell persistence and improved tumor treatment.

ACLY-dependent acetyl-CoA generation is also implicated in T cell responses to microbiota-derived metabolites. Thus, Luu et al. found that supplementing T_H_17-cultured T cells with the short-chain fatty acid pentanoate induced the secretion of the immunomodulatory cytokine IL-10 in an ACLY-dependent manner. At the same time, pentanoate suppressed the induction of T_H_17-associated genes, likely through its inhibitory effect on histone deacetylases. Pentanoate supplementation enhanced glycolysis, acetyl-CoA production, and histone acetylation at the *Il10* promoter (108).

Relatively little is known as to whether ACLY is regulated by post-translational modifications in T cells. In a nuclear phosphoproteome study of the Kit225 T-cell leukemia cell line, ACLY was a clear candidate at mediating IL-2-driven T cell proliferative responses. The enzyme was phosphorylated at Ser455 upon IL-2 activation *via* the phosphatidylinositol 3-kinase (PI3K)/Akt pathway. In addition, this was the first study to show that ACLY Ser646 can be phosphorylated in T cells, although the relevance of this phosphorylation site still needs to be identified. ACLY inhibition resulted in a reduction of histone acetylation and impaired IL-2-induced T-cell growth. Despite the coexistence of several acetyl-CoA generating enzymes in the nucleus, such as PDC and ACSS2, ACLY appeared to be the principal source of nuclear acetyl-CoA in Kit225 T cells (109).

### The Role of ACLY in B Lymphocytes

To generate a proper adaptive response, the bioenergetic demands placed on B lymphocytes upon their activation increase considerably. In consequence to antigen challenge, quiescent B lymphocytes transit to highly activated states, proliferate and differentiate to antibody-secreting plasma cells (110, 111). During this process, B lymphocytes require large amounts of lipid and cholesterol, accompanied by high rates of aerobic glycolysis, as observed in many cancer and other immune cells (111, 112).

The role of ACLY in B cells is still poorly understood and remains an open topic of investigation. Notwithstanding, ACLY activity was reported to be crucial in regulating glucose-dependent *de novo* lipogenesis and proliferation as well as the acquisition of plasma cell-specific markers CD138 and Blimp-1 in splenic B lymphocytes following LPS stimulation or B-cell receptor cross-linking. These treatments also elicited PI3K-dependent phosphorylation of ACLY at Ser455. Moreover, CH12 B lymphoma cells, which are employed as a model for studying IgM production in B cells, showed a reduced antibody secretion upon ACLY inhibition. In summary, these results place ACLY as a key regulator of phenotypic changes that influence plasma cell differentiation (113). Regarding the role of acetyl-CoA in epigenetic remodeling of activated B cells, a recent study of chromatin decompaction in murine B cells activated with LPS and IL-4 found substantial increases of global histone acetylation, predominantly at histone H4 residue Lys16, histone H3 Lys14, and Lys27 (114). This was suggested to be, at least in part, due to increased production of acetyl-CoA, supported by increased protein expression of ACLY, as inhibition of ACLY by Medica 16 decreased H3 Lys27 acetylation.

Based on these results, further studies are necessary to fully elucidate the apparent important role of ACLY in modulating epigenetic changes in B lymphocytes.

## Discussion

It has become evident that metabolic and epigenetic changes are tightly inter-regulated during immune responses. ACLY control of fatty acid metabolism and histone acetylation through the provision of acetyl-CoA seems to be a key regulatory element, as supported by multiple recent studies (summarized in **Table 1**). Nonetheless, while our understanding toward the role of ACLY in the immune system advanced considerably in recent years, many open questions remain. Many conclusions about the role of ACLY in modulating immune responses were based on the use of ACLY pharmacological inhibitors, which may have off target effects. As such, further studies employing genetic models of ACLY deficiency in specific immune cell types should be employed to study the functions of ACLY, especially in the *in vivo* setting. Moreover, advanced mass-spectrometry analyses can give deeper insights into stoichiometry and dynamics of acetylation of individual histone lysine residues during immune responses and their regulation by substrate availability. Mechanistically, it is unclear whether ACLY may act at specific nuclear loci associated with ACLY-dependent genes and if it does, what drives this association. In addition, the role of ACLY in the immune regulation of other immune cells, such as neutrophils or eosinophils, still needs to be explored. Furthermore, the contribution of other acetyl-CoA producing enzymes to epigenetic regulation has also attracted interest. It would be noteworthy to study the role of these ‘moonlighting’ enzymes in more detail.

**Table 1:** Summary of ACLY roles in immune cells.

Cell type	Regulation	Physiological outcome
**Innate immune system**		
Macrophages	Metabolic	Pharmacological inhibition or mRNA silencing of ACLY results in a reduction in nitric oxide (NO), reactive oxygen species (ROS), and prostaglandin E_2_ (PGE_2_) release upon LPS stimulation (71) Myeloid-specific ACLY deficiency stabilizes atherosclerotic plaque phenotype (82)
	Epigenetic	Glucose-derived, ACLY-dependent acetyl-CoA increases histone acetylation in LPS-stimulated BMDM (78) ACLY provides acetyl-CoA in IL-4-treated BMDM for histone acetylation in a gene-specific manner (80) ACLY is not required for histone acetylation in IL-4 treated human primary macrophages (72)
DC	Metabolic	ACLY-dependent *de novo* lipogenesis supports DC maturation (92)
NK	Epigenetic	Inhibition of ACLY in NK cells causes reduced IFNγ production and granzyme B expression (23)
**Adaptive immune system**		
T cells	Metabolic	Depletion or inactivation of ACLY impairs IL-2- mediated T-cell growth (109)
	Epigenetic	Acetyl-CoA is required for IFNγ production by activated T_H_1 cells (9) Acetyl-CoA generated by ACLY boosts rapid memory responses in CD8^+^T cells (104, 105) Pentanoate supplementation enhanced glycolysis, acetyl-CoA production and histone acetylation at the *Il10* promoter in an ACLY-dependent manner (108) ACLY enhances histone acetylation and regulates cell cycle genes in IL-2-treated cells (109)
B cells	Metabolic	ACLY dependent lipogenesis drives key phenotypic changes required for plasma cell differentiation (113)
	Epigenetic	ACLY inhibition reduced histone acetylation upon LPS and IL-4 treatment in murine B cells (114)

## Author Contributions

MD: writing of the original draft. BB, DN: editing and writing of the final draft. All authors contributed to the article and approved the submitted version.

## Funding

We acknowledge the financial support of Deutsche Forschungsgemeinschaft: SFB 1039 (Teilprojekt A05, B04), NA 429/2-2.

## Conflict of Interest

The authors declare that the research was conducted in the absence of any commercial or financial relationships that could be construed as a potential conflict of interest.

## Abbreviations

ACC, acetyl-CoA carboxylase; ACLY, ATP-citrate lyase; ACSS2, acyl-CoA synthetase short-chain family member 2; BMDM, bone marrow-derived macrophages; CoA, coenzyme A; DC, dendritic cells; FAS, fatty acid synthase; Gpd2, glycerol 3-phosphate dehydrogenase; HCMV, human cytomegalovirus; IFNγ, interferon γ; IL, interleukin; LDHA, lactate dehydrogenase A; LPS, lipopolysaccharides; LXR, liver X receptor; MDH, malate dehydrogenase; NK, natural killer; NO, nitric oxide; OXPHOS, oxidative phosphorylation; PAF, platelet activating factor; PDC, pyruvate dehydrogenase complex; PGE_2_, prostaglandin E_2_; PI3K, phosphatidylinositol 3-kinase; PRR, pattern recognition receptor; ROS, reactive oxygen species; SREBP, sterol regulatory element-binding protein; TCA, tricarboxylic acid; TLR, toll-like receptor; TNFα, tumor necrosis factor α.
